# Aniline Dyes and Tumours of the Bladder

**Published:** 1904-09-03

**Authors:** 


### ANILINE DYES AND TUMOURS OF THE BLADDER.

Frankfort[1] holds that these tumours are chiefly observed in factories where aniline, its homologues and allied substances are prepared. After many observations which extended to English factories he came to the conclusion that the shortest time for a patient to develop the tumours was after working for nine years. Out of 23 cases five patients developed papillomata (two of them afterwards becoming malignant) one developed a sarcoma and 17 suffered from carcinomata. Many years ago, in his surgical lectures, Professor Ogston used to observe that there appeared to be a special liability to vesical tumours among those whose occupation brought them into contact with aniline dyes.

[1] Surgical Congress, Berlin, July 10, 1904.

